# Pre-sleep Protein Supplementation Affects Energy Metabolism and Appetite in Sedentary Healthy Adults

**DOI:** 10.3389/fnut.2022.873236

**Published:** 2022-04-28

**Authors:** Yingying Hao, Xingchen Li, Zheng Zhu, Zhen-Bo Cao

**Affiliations:** ^1^Shanghai Frontiers Science Research Base of Exercise and Metabolic Health, Shanghai University of Sport, Shanghai, China; ^2^School of Kinesiology, Shanghai University of Sport, Shanghai, China

**Keywords:** protein, supplementation, pre-sleep, sedentary, healthy, adults

## Abstract

**Purpose:**

To assess the acute effect of pre-sleep protein supplementation combined with resistance exercise on energy metabolism (including 24-h total energy expenditure (TEE), sleep energy expenditure (SEE), basal energy expenditure (BEE), glycolipid oxidation, and appetite of sedentary adults.

**Methods:**

A total of thirty-one sedentary participants completed this randomized, double-blind, crossover study. Participants completed the following 24-h experimental conditions in random order in the Human Calorimeter chamber: (1) 40-g protein supplementation with dinner before a nighttime resistance exercise, and followed by pre-sleep placebo intake (PRO-PLA); (2) placebo intake with dinner before a nighttime resistance exercise, and followed by pre-sleep 40-g protein supplementation (PLA-PRO); and (3) placebo supplementation both with dinner and pre-sleep combined with a nighttime resistance exercise (PLA). Subjective appetite score before breakfast the next day was evaluated using the visual analog scale.

**Results:**

The SEE values were significantly higher by a mean of 21.7 kcal and 33.3 kcal in PRO-PLA (318.3 ± 44.3 kcal) and PLA-PRO (329.9 ± 45.2 kcal), respectively, than in PLA (296.6 ± 46.6 kcal). In addition, the SEE values for PLA-PRO was also significantly higher by 11.6 kcal than that for PRO-PLA. Further, the fullness the next morning was significantly higher by 30.8% in PLA-PRO (43.9 ± 23.5 mm) than in PLA (33.5 ± 26.6 mm). These effects remained after adjustment for 24-h energy intake.

**Conclusion:**

Pre-sleep protein supplementation combined with resistance exercise can significantly increase the SEE and fullness in the next morning, indicating a possible strategy to improve sleep energy metabolism in the sedentary population.

## Introduction

Pre-sleep food intake is a largely unexplored part of nutrient timing research but may be a key factor in regulating the effects of different macronutrients on body composition, metabolism, and satiety (1). However, over the past decades, it was thought that large meals or the majority of daily nutrients close to nighttime sleep should be limited and/or avoided because it would increase the likelihood of weight gain (2, 3) and negatively impact health and body composition. Ultimately, this may increase the risks for cardiometabolic diseases such as obesity and diabetes (3–5). However, several studies have shown that pre-sleep food intake can positively enhance metabolic health and body composition when food choices are altered to small, nutrient-dense, low-energy and/or single macronutrients foods (<200 kcals) (1, 3, 6–10).

Additionally, pre-sleep protein supplementation within 30 min of sleep has been considered a new nutrient timing strategy in recent years (11). In recent research, pre-sleep protein supplementation has been shown to improve overnight muscle protein synthesis that results in improved skeletal muscle adaption to exercise (12–15). After a single bout of evening resistance exercise, muscle protein synthesis rates during overnight sleep were approximately 22% higher in recreational athletes who consumed protein prior to sleep than in those who ingested a placebo drink (16). Some studies have also investigated the impact of this nutritional strategy on non-muscular parameters such as metabolism and appetite. Madzima et al. found that 48-g pre-sleep protein supplementation with resistance exercise elicited favorable changes in morning basal metabolic rate (RMR) in active women while the placebo did not (11), and Ormsbee et al. found that pre-sleep acute consumption of casein reduces appetite and increases fullness the next morning in obese women (9). In contrast, Kinsey et al. reported pre-sleep casein protein supplementation did not affect fat metabolism and rest energy expenditure (EE) or suppress appetite in obese men with hyperinsulinemia (17).

Almost all physiological and behavioral functions of the body exhibit oscillations during different time periods, mainly in 24-h cycles (18). Additionally, the reduction of resting metabolic rate during sleep, considered as a 6–8 h new nutritional window, is one of the important factors for weight gain caused by pre-sleep food consumption (19). Metabolic adaptations reflect changes in EE (20). However, no studies have evaluated the effects of pre-sleep protein supplementation on total 24-h energy expenditure (TEE) and sleep energy expenditure (SEE) because of the difficulty of accurately measuring the SEE (21).

Research indicates that pre-sleep protein supplementation could have an anabolic effect on overnight muscle protein synthesis despite the relatively high amount of protein consumed earlier in the day (12). This suggests that we need to consider the “total dose effect” and “time effect,” that is, if pre-sleep protein ingestion has an impact on energy metabolism, it should be determined whether this effect is caused by the total amount of protein in a day or the timing of consumption. To the best of our knowledge, there is only 1 study controlling protein timing that found no significant difference in whole-body substrate utilization between daytime and pre-sleep protein supplementation (22), and thus, the finding of promising metabolic outcomes has limited generalizability.

This study aimed to examine the acute effect of pre-sleep protein on TEE, SEE, BEE, glycolipid oxidation, and appetite of sedentary adults. We hypothesized that pre-sleep protein supplementation combined with resistance exercise will be effective in improving energy metabolism and appetite in sedentary healthy individuals.

## Materials and Methods

### Study Design and Participants

This randomized, three-group, double-blind crossover study enrolled 31 sedentary, healthy, weight-stable adults (11 men and 20 women) using the social application, WeChat^1^ or leaflets distributed on university campuses in Shanghai. The inclusion criteria were age (1) 20–39 years and (2) not overweight and obese, inactive, and sedentary (i.e., current sedentary behavior: ≥8 h/d and not meeting the physical activity recommended guideline of ≥150 min/week of moderate-intensity or ≥75 min/week of vigorous-intensity exercise for at least 3 months) (23). The exclusion criteria were (1) smoking, (2) pregnancy, (3) change in body weight >2 kg in the past 3 months, (4) metabolic syndrome, (5) musculoskeletal injury within the past 6 months with contraindications for physical activity, (6) systolic/diastolic blood pressure >140/90 mmHg, (7) intake of any medication or supplement that affects glucose and lipid metabolism, (8) acute illness, unstable chronic conditions, neurological disorders, and cognitive disorders, and (9) allergy to milk and protein-rich foods.

**FIGURE 1:**
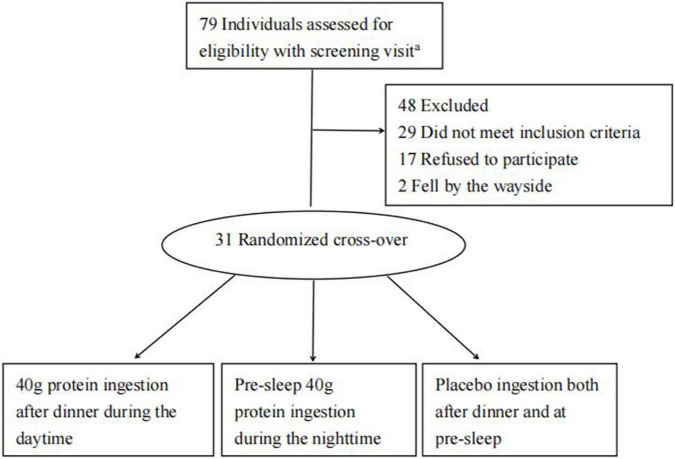
Participant inclusion flowchart. *^a^*There were 201 people who contact us by network in response to the E-recruitment poster, but only 79 were considered appropriate for further standard screening.

The present study asked participants to refrain from taking any nutritional supplements for at least 3 months prior to and throughout the duration of study. The recruitment, randomization, and follow-up are shown in Figure 1.

This study was approved by the Ethics Committee of Shanghai University of Sport (102772020RT025) and was registered in the Chinese Clinical Trial Registry (ChiCTR2100041871). All participants provided informed consent.

### Trial Protocol

This study was conducted between January 15 and August 31, 2021. Participants visited the laboratory four times: once for a preliminary test and three times for crossover experiments. They completed the following 24-h experimental conditions in random order: (1) 40-g protein supplementation with dinner before a nighttime resistance exercise, and followed by pre-sleep placebo intake (PRO-PLA); (2) placebo intake with dinner before a nighttime resistance exercise, and followed by pre-sleep 40-g protein supplementation (PLA-PRO); and (3) placebo supplementation both with dinner and pre-sleep combined with a nighttime resistance exercise (PLA). There was at least 7–14-day washout between conditions to eliminate potential carryover effects.

### Pre-experimental Stage

At least 7 days before the experiment officially started, a familiarization visit took place in the laboratory. Height and weight were measured using HK600-ST (Hengkang Jiaye Technology Co., Ltd., ShenZhen, China). The participants also underwent a body composition test *via* dual-energy X-ray absorptiometry (Lunar Prodigy: GE Healthcare, Chicago, IL, United States), followed by a maximal aerobic-capacity test (VO_2max_) using a modified version of the Bruce protocol on a treadmill, with the gas collected using a Cosmed K5 metabolic gas analyzer (Cosmed, Rome, Italy).

At 3 days before each session, the participants were asked to wear with an accelerometer (GT3X+: ActiGraph, Pensacola, FL, United States) in their hip on the right anterior superior iliac spine to monitor the time spent on sedentary behavior, and moderate-to-vigorous (MVPA) in daily life and were asked to refrain from structured moderate-to-vigorous physical activity, caffeine, and alcohol. Concurrently, the participants recorded their daily diets for the first and second day and copied the diet before each subsequent intervention condition. At 1 day before each intervention, the participants began to take standard meals.

### Experimental Stage

**FIGURE 2:**
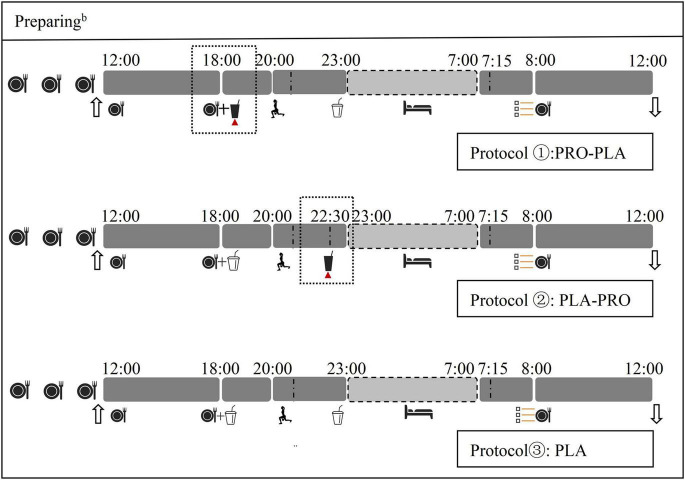
Study protocol for the experimental conditions. *^b^*The day before experiment; 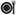 Standard meal 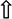 Enter the HC chamber 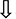 Out of the HC chamber 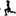 Resistant exercise 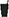 Protein supplementation 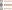 Visual analog scale (VAS) test 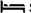 Sleep 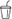 Placebo.

The participants underwent three 24-h experimental conditions inside the Human Calorimeter (HC) chamber (3.85 m width × 2.85 m depth × 2.5 m height; FHC-20S: Fuji Medical Science Co, LTD, Chiba, Japan). Each 24-h experimental condition (Figure 2) included a 6-h sedentary period (12:00–18:00), a 5-h intervention period (18:00–23:00), an 8-h sleep period (23:00–7:00 next day), a 45-min basal metabolic period on the next day (7:15–8:00: BEE), and a 4-h sedentary period on the next day (8:00–12:00). Moreover, subjective appetite score before breakfast the next day was evaluated using a visual analog scale (VAS). The sequence of experimental condition is based on numbers randomly generated by the computer. The study protocol is detailed in Figure 2.

In the three conditions, except for the resistance exercise and basal metabolic rate test, participants maintained sedentary behavior only while awake, including during reading, writing, listening to music, watching TV, and operating computer in office. The research staff directly supervised the participants for compliance with the protocol. Female participants completed these conditions in the follicular phase of their menstrual cycle to reduce variation in metabolic functions (24).

### Exercise Training

The exercise period included a 5-min warm up, 35-min resistance exercise-based lower limb exercise, and 10-min cool down. The activities mainly involved high squats, squat pulses, lunge, lunge pulses, close squats, squat walk, squat rocks, curtesy pulses, pivot lunge, glide pivot lunge, pass through, and lateral and sumo pulses. Each exercise performed at 8–12 repetitions with 15–30 s rest per set according to the sequence above, total three sets. All exercises were performed with body weight or dumbbells. The dumbbell loads for males and females were 10 and 5 kg, respectively. This load was determined based on 8-12RM training principles (25) in the preliminary experiment.

### Meals and Protein Supplementation

#### Meals

The participants consumed standard breakfast (8:00–8:20), lunch (12:00–12:30), and dinner (18:00–18:30) during the pre-experimental and experimental stage. All standard meals were personalized to meet daily estimated energy requirements calculated using the Schofield equation (1.4 physical activity factor) (26). The energy intake provided by breakfast, lunch, and dinner accounted for approximately 30%, 40%, and 30% of the daily energy requirements, respectively. The meals provided by research staff were standardized for all three conditions for all participants. Total energy intake was estimated from nutrient composition table of food package and Chinese Food Composition (2nd edition) (27). In addition, the participants were given 1.0 g/kg/day protein *via* the standardized diet according to the Chinese Dietary Nutrient Reference Intakes by the Chinese Nutrition Society (28). This was evenly distributed among the three daily meals.

#### Protein Supplementation

Additional supplementation of 40-g protein was provided during PRO-PLA and PLA-PRO, as based on previous recommendations (12). In PRO-PLA and PLA-PRO, participants consumed 52-g strawberry flavored casein (CAS protein content: 40 g; 183.0 kcals; 0.9 g fat; 3.3 g carbohydrate (CHO); 46.8 mg sodium; 1211.6 mg calcium). In PLA, participants consumed the placebo (Propel Zero™: The Gatorade Company, Chicago, IL, United States; 2.4 g; 0 kcals) with the same flavor (strawberry) and powder texture as the control supplementation.

Casein and the placebo supplementation were pre-mixed with 500 ml water in a dark-colored shaker bottle, which was provided to participants during the specified time periods mentioned above. Participants consumed each supplement under the supervision of researchers.

### Energy Expenditure and Substrate Oxidation of Energy Metabolism

Energy expenditure indicators as main outcome included TEE, SEE, and BEE and were measured in a humidity curing (HC) chamber. The human HC contains a wash stand, bed, toilet, desk with chair (29). The temperature and relative humidity of incoming fresh air were maintained at 25.0°C (± 0.5°C) and 50.0% (± 3.0%), respectively. The sample air was dehumidified using a gas-sampling unit (SCC-C, ABB Corp, Japan) and analyzed using a mass spectrometer (Prima PRO, Thermo Fisher Scientific, Cheshire, United Kingdom), and the system was calibrated using standard gas every two weeks (30). The chamber software allows the measurement of EE with high-time resolution by detecting changes in activity level (31). Oxygen consumption (VO_2_) and carbon dioxide production (VCO_2_) were calculated by using the formula of Henning et al (32). The fat and CHO oxidation rate were calculated using equation as follows (33):


$$\text{Fat oxidation rate (g/min)}=1.695\times {\text{VO}}_{2}\,\left(\text{L/min}\right)-1.701\times {\text{VCO}}_{2}\,\left(\text{L/min}\right)$$

$$\text{CHO oxidation rate (g/min)}=4.585\times {\text{VCO}}_{2}\,\left(\text{L/min}\right)-3.226\times {\text{VO}}_{2}\,\left(\text{L/min}\right)$$

### Appetite

Appetite as secondary outcome was assessed using a validated VAS (34). Briefly, the VAS was a 100 mm horizontal scale from 0 to 100 with different scale positions representing different levels of appetite, with appetite increasing gradually along the horizontal scale (0 = “no appetite at all” and 100 = “extreme appetite”). Participants assessed their current subjective feelings of hunger, fullness, desire to eat and perspective consumption by putting a vertical line along the horizontal scale (17). The total appetite score was calculated as follows (35): appetite score = [perspective consumption + desire to eat + hunger + (100 - fullness)] / 4.

### Statistical Analyses

Sample-size calculations were based on a previous study using similar methodology in sedentary population (8), assuming a 13% increment in total 24-h EE for pre-sleep protein ingestion. Therefore, we estimated that 22 paired participants would be required to achieve 80% power to detect the effect size (Cohen d = 0.40) in the main outcome, with a two-sided alpha of 0.05. With consideration for potential withdrawals, 31 participants were enrolled.

The data were first tested for normality using standardized skewness and kurtosis values. Normally distributed data were presented as the mean and standard deviation, while non-normally distributed data were presented as the interquartile range. Linear mixed models (LMMs) and generalized LMMs were used to assess the differential impacts of the intervention conditions on all outcome indicators for normally and non-normally distributed data, respectively (30). Model 1 was adjusted for potential confounders explaining main outcome variance (age, sex, %fat, relativeVO_2max_, sleep quality, time of sedentary behavior and moderate and vigorous-intensity physical activity before each intervention condition, and resistance EE under each condition) and period effects (condition order). Model 2 was additionally adjusted for 24-h total energy intake (including energy from the standard meals intake and protein supplementation). If there was a main effect of the intervention condition, a Bonferroni corrected test was applied to *post-hoc* analysis. All statistical analyses were performed using SPSS Statistics version 20.0 (IBM Corp).

## Results

### Participant Characteristics

The participant characteristics are summarized in Table 1. The accelerometer data under free-living conditions confirmed the participants’ physical inactivity (MVPA, 19.5 ± 12.6 min/day) and sedentary (sitting time, 625.9 ± 102.5 min/day).

**TABLE 1:** Participant characteristics.

Variables	All (*n* = 31)	Female (*n* = 20)	Male (*n* = 11)
Age (years)	25.0[21.0,26.0]	25.0[21.0, 27.5]	24.2 ± 4.6
Height (cm)	167.1 ± 8.6	163.2 ± 6.6*	174.1 ± 7.4
Weight (kg)	61.3 ± 9.2	57.1 ± 7.4*	69.0 ± 7.0
BMI (kg/m^2^)	21.9 ± 1.8	21.4 ± 1.8*	22.7 ± 1.5
Percent body fat (%)	25.3 ± 8.3	30.6 ± 4.3*	15.9 ± 4.5
**Metabolic and cardiovascular risk factors**			
Fasting glucose (mmol/L)	4.93 ± 0.25	4.93 ± 0.25	4.89 ± 0.27
Fasting insulin (pmol/L)	48.0 ± 22.3	54.7 ± 24.0*	35.2 ± 10.3
Fasting triglyceride (mmol/L)	0.96 ± 0.4	0.95 ± 0.5	0.98 ± 0.3
Total cholesterol (mmol/L)	4.29 ± 1.05	4.25 ± 1.11	4.38 ± 1.59
Systolic blood pressure (mmHg)	113.5 ± 10.7	113.5 ± 10.7*	121.1 ± 8.9
Diastolic blood pressure (mmHg)	70.5[66, 75.5]	68.1 ± 5.7*	73[72, 88]
**Aerobic fitness**			
Absolute VO_2max_(L/min)	2.4[2.1, 2.3]	2.2 ± 0.3*	3.7 ± 0.6
Relative VO_2max_(mL/kg⋅min)	40.7[35.7, 49]	38.1 ± 4.7*	53.1 ± 7.9
**Physical activity level**			
Sedentary time (min/day)	625.9 ± 102.5	623.4 ± 95.3	630.5 ± 119.3
MVPA time (min/day)	19.5 ± 12.6	18.1 ± 6.8	22.2 ± 9.0
**Sleep quality**			
sleep quality index	4.4 ± 2.4	3.8 ± 2.4	5.5 ± 2.0

*Normally distributed data are represented by mean ± standard deviation (Mean ± SD), and non-normal distribution data are represented by the median and interquartile range.*

P-values were generated using independent two-sample t-test; *P < 0.05 vs. Male.

### Physical Activity Levels and Energy Intake

Table 2 shows the physical activity level during the pre-experimental stage and energy intake during the experimental stage. The physical activity level before each intervention had no significant difference among the conditions.

**TABLE 2:** Physical activity level before each intervention and energy intake during the experimental stage.

Variables	PLA	PRO-PLA	PLA-PRO	*p*
**Physical activity level (min/day)**				
Sedentary time_–GT3X+_	610.1 ± 116.4	623.7 ± 100.2	666.0[549.6, 713.7]	0.314
MVPA time_–GT3X+_	16.0[10.6, 22.7]	19.2 ± 11.8	14.3[7.7, 22.7]	0.796
**Energy intake (kcal)**				
Meal intake	2092.1 ± 330.9	2092.6 ± 332.4	2097.8 ± 337.8	0.997
Protein_–supplementation_	0	183.0	183.0	
Protein_–meals_	253.5 ± 41.1	253.8 ± 41.0	253.9 ± 41.5	0.999
CHO_–meals_	1133.4 ± 239.4	1131.1 ± 233.3	1136.1 ± 230.6	0.995
Fat_–meals_	705.2 ± 152.5	707.7 ± 166.9	707.7 ± 166.0	0.998

*24-h total energy intake = energy from meals intake +energy from protein supplementation.*

Analysis of standard meals showed meal intake was similar between conditions, with a mean intake of 2094 kcal/day (female = 1905 kcal/day; male = 2438 kcal/day), with 54.2% energy from CHO, 33.7% energy from fat, and 12.1% energy from protein. The 24-h total energy intakes were 2276 kcal/day, 2281 kcal/day, and 2092 kcal/day for PRO-PLA, PLA-PRO, and PLA, respectively.

### Total Energy Expenditure, Sleep Energy Expenditure, and Basal Energy Expenditure

**FIGURE 3:**
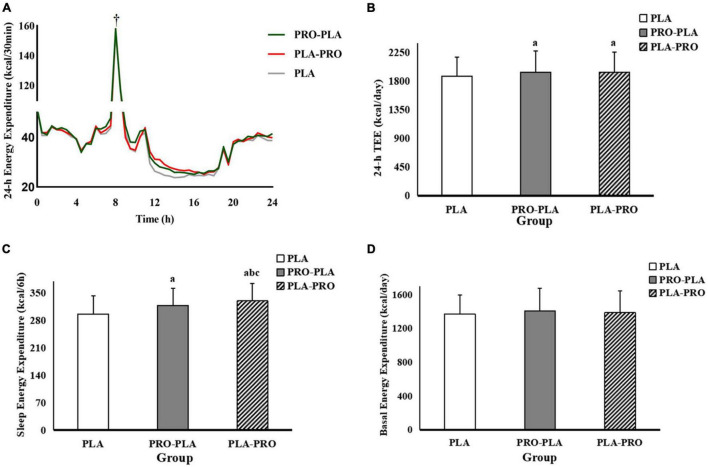
The 24-h Continuous dynamic energy expenditure **(A)**, total energy expenditure (TEE, **B**), sleep energy expenditure (SEE, **C**), and basal energy expenditure (BEE, **D**) in PRO-PLA, PLA-PRO, and PLA. ^†^Peak energy expenditure caused by resistance exercise. *^a^p* < 0.05 PLA-PRO, PRO-PLA VS PLA, *^b^p* < 0.05 PLA-PRO VS PRO-PLA, adjusted for age, sex, %fat, relative VO_2max_, sleep quality, the time of sedentary behavior and moderate and vigorous intensity physical activity in the pre-experiment stage, resistance energy expenditure under each condition (model 1); *^c^p* < 0.05 PLA-PRO VS PRO-PLA, additionally adjusted for 24-h total energy intake based on model 1 (model 2).

The TEE, SEE, and BEE are shown in Figure 3. The specific values can be found in Supplementary Appendix Table 1. The values of TEE during PRO-PLA (1945 ± 331 kcal/day) and PLA-PRO (1939 ± 317 kcal/day) were significantly higher than those during PLA (1883 ± 293 kcal/day). All these above significant effects disappeared after adjustment for 24-h total energy intake. SEE was higher by 21 and 33 kcal in PRO-PLA (318 ± 44 kcal) and PLA-PRO (330 ± 45 kcal), respectively, than in PLA (297 ± 47 kcal). In addition, SEE was significantly higher by 12 kcal in PLA-PRO than in PRO-PLA. These effects remained after adjustment for 24-h energy intake. There was no significant difference in BEE among the intervention conditions.

### Carbohydrates and Fat Oxidation

**FIGURE 4:**
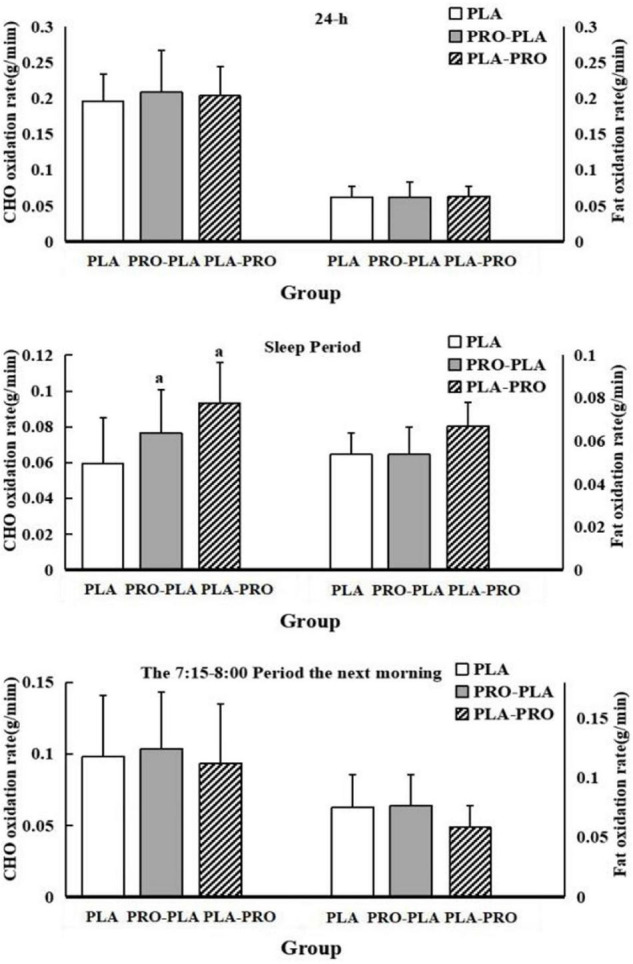
Carbohydrate (CHO) and fat oxidation rates within 24 h, during sleep, and during the basal metabolic period in PLA, PRO-PLA, and PLA-PRO (unit: g/min). *^a^p* < 0.05 PLA-PRO, PRO-PLA VS PLA, adjusted for age, sex, %fat, relative VO_2max_, sleep quality, the time of sedentary behavior and moderate and vigorous intensity physical activity in the pre-experiment stage, resistance energy expenditure under each condition (model 1); additionally adjusted for 24-h total energy intake based on model 1 (model 2).

The CHO and fat oxidation rates within 24 h, during sleep, and during the basal metabolic period are shown in Figure 4. The specific values are shown in Supplementary Appendix Table 1. There was no significant difference in fat oxidation among the conditions in all periods evaluated. The CHO oxidation rates were also not significantly different, except for the CHO oxidation rate during sleep. As shown in Figure 4, the CHO oxidation rate during sleep was significantly higher by 33% during PRO-PLA (0.08 ± 0.02 g/min) and PLA-PRO (0.08 ± 0.02 g/min) than during PLA (0.06 ± 0.03 g/min). The differences disappeared after adjustment for total 24-h energy intake.

### Appetite

**FIGURE 5:**
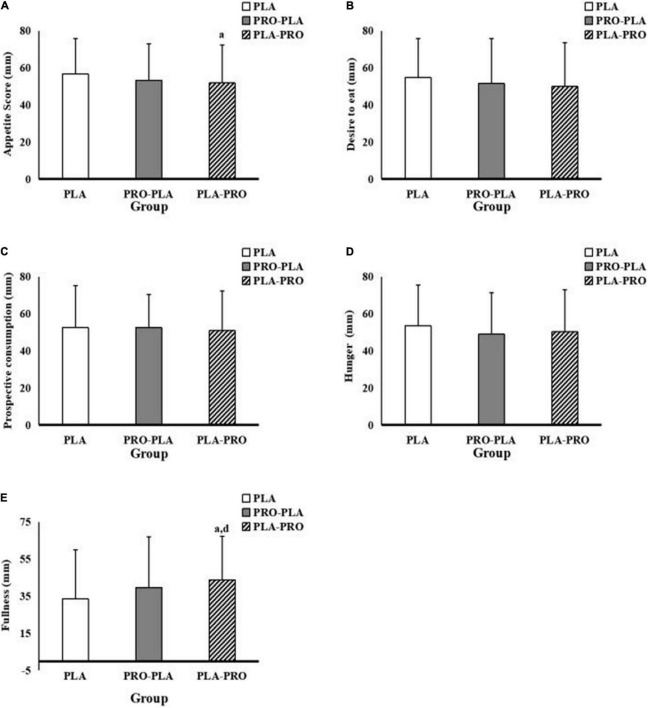
Appetite scores in PLA, PRO-PLA, and PLA-PRO (unit: mm). **(A)** The appetite score. **(B)** Desire to eat. **(C)** Prospective consumption. **(D)** Hunger. **(E)** Fullness. *^a^p* < 0.05 PLA-PRO VS PLA, adjusted for age, sex, %fat, relativeVO_2max_ sleep quality, the time of sedentary behavior and moderate and vigorous intensity physical activity in the pre-experiment stage, resistance energy expenditure under each condition (model 1); *^d^p* < 0.05 PLA-PRO VS PLA, additionally adjusted for 24-h total energy intake based on model 1 (model 2).

The subjective appetite scores are shown in Figure 5. The specific values can be found in Supplementary Appendix Table 1. Overall, the total appetite score is significantly lower by 9.6% (*p* = 0.046) in PLA-PRO (51.9 ± 20.5) than in PLA (56.9 ± 19.2) in model 1. However, there was no significant difference among the conditions in model 2. In addition, fullness was significantly higher by 30.8% in PLA-PRO (43.9 ± 23.5) than in PLA (33.5 ± 26.6) in model 1 (*p* = 0.042) and 2 (*p* = 0.038).

## Discussion

The present study found that pre-sleep protein ingestion significantly increases TEE and CHO oxidation rates, but it has no significant effect on BEE in the next morning and fat oxidation rate. These results were affected by total 24-h energy intake. As expected, pre-sleep protein intake significantly increased SEE and fullness in the next morning and significantly reduced total appetite score in the next morning. Interestingly, these results were independent of total 24-h energy intake.

TEE should be accurately assessed to determine the effect of pre-sleep protein intake on energy balance. However, no study has investigated the influence of pre-sleep protein intake on TEE, possibly due to equipment limitations. To our best knowledge, this study is the first to show that pre-sleep protein supplementation combined with resistance exercise can increase TEE. Further, the increment in TEE is not independent of total energy intake, regardless of timing of protein intake. Bray et al. found that the effect of protein overfeeding on changes in TEE were significantly related to dietary protein intake (*r* = 0.50, *p* = 0.020) but not to dietary energy intake (*r* = 0.14, *p* = 0.82) (36). Therefore, we speculated that increases in TEE may be related to the protein being utilized to stimulate muscle and whole-body protein synthesis in the body.

A previous research evaluated EE according to the residual mass calculated as total mass minus brain mass, skeletal muscle mass, bone mass, and adipose tissue mass from the DXA model (37) and found a significant positive correlation to protein intake. This is consistent with the idea that the increasement of energy utilization to catabolize the excess protein caused by the liver and/or kidney, which are important sources of energy utilization in the residual mass (36). Traditionally, energy intake is limited in the late evening as metabolism is believed to slow down during sleep (19, 38) and may therefore promote energy storage. However, although metabolism has been shown to decrease at night (19), there is no scientific evidence showing that this applies to pre-sleep protein consumption (1). Dietary proteins have a greater thermic effect than other macronutrients (39–43), and there have been findings that higher daily protein intake better attenuates the typical decrease in sleep-time EE than intake of other macronutrients and lower-protein diets (40, 44).

In addition, data also suggest that the amount of protein intake throughout the day impacts the reduction of SEE in women (44). Consistently, the findings of the subgroup analysis in the present study show that pre-sleep protein supplementation significantly increased the SEE of sedentary females (PLA-PRO vs. PLA: 302.8 ± 24.7 kcal/6 h vs. 276.0 kcal/day ± 30.1 kcal/6 h, *p* < 0.001). Overall, the present study found that protein supplementation at night and pre-sleep can significantly increase SEE, with pre-sleep protein supplementation being more effective. Thus, it can be expected that our findings have a “time effect.” This suggests that protein supplementation can effectively maintain or even improve the metabolic capacity during sleep when it is consumed closer to the sleep period. At present, traditional nutritional and exercise interventions related to metabolic health are mostly implemented during daytime. Our findings provide baseline evidence that combining daytime behavioral changes with nighttime nutrition supplementation may increase the efficiency of intervention and better improve energy metabolism.

The present study confirmed that pre-sleep protein supplementation had no significant effect on RMR in the next morning, that is, there is no sustained effect on the metabolic response in sedentary healthy adults. This result is consistent with most findings that 30-g casein intake at pre-sleep does not significantly affect the RMR in the next morning in active healthy women (14, 22, 45, 46) and in obese women and men (8, 9, 17). In contrast, Madazima et al. observed substantial changes in RMR in lean, physically active men the next morning (1) when the pre-sleep protein supplement was 30 g. The findings of the subgroup analysis in the present study show that pre-sleep protein supplementation influenced the BEE of sedentary males (PLA-PRO vs. PLA: 1627 kcal/day vs. 1569 kcal/day), but not of sedentary females (PLA-PRO vs. PLA: 1253 kcal/day vs. 1267 kcal/day). This suggests that pre-sleep protein supplementation has sex-specific effects on BEE. The relative max oxygen uptake (VO_2max_) and lean mass of males is significantly higher than that of females in the current study. Lean mass is related directly to total daily REE (47); thus, we speculate that sex-specific effects may be explained by higher levels of lean mass in males.

Interestingly, pre-sleep protein supplementation did not hinder fat oxidation and increased CHO oxidation during sleep period. Most studies have only focused on the effect of pre-sleep protein supplementation on fat oxidation, and there are no data on CHO oxidation. The findings are consistent with those of Ormsbee et al. who found that pre-sleep 30-g protein intake combined with exercise for 4 weeks did not alter fat oxidation in young obese women (9). Similarly, pre-sleep protein supplementation did not stimulate nor suppress fat metabolism in resistance-trained women (22) and young obese women (8). However, Madzima et al. found that pre-sleep ingestion of casein protein increases morning fat oxidation in active college men (1). In contrast, some studies reported that pre-sleep whey protein or other macronutrient supplementation (e.g., carbohydrate snacks) can significantly reduce fat oxidation (48, 49). Macronutrient intake leads to an increase in insulin concentration that can markedly lower fat oxidation and shift substrate utilization toward carbohydrate oxidation (50). The slow-digesting nature of casein may result in a blunted insulin response (22), thereby leading to reduced inhibition of fat oxidation and sustained increase of CHO oxidation.

Current evidence indicates that pre-sleep acute consumption of casein reduces appetite and increases fullness the next morning in overweight and obese people (8, 9) but not in physically active people (1, 46) and older individuals (15, 51). Our study complements and extends the previous findings by showing that pre-sleep acute consumption of casein protein in sedentary healthy adults leads to reduced total appetite score and increased fullness in the next morning. The increase in morning satiety in overweight and obese participants was suspected to be a compensatory response to the extra nighttime calories. However, our study found that the increase in fullness caused by pre-sleep protein intake is independent of total energy intake. Collectively, these results suggest that the effects of pre-sleep protein supplementation on the fullness cannot solely be explained by an increase in energy intake, and other participant characteristics may also be involved.

The first strength of the present study is that we controlled the timing of protein intake to understand whether the effect of pre-sleep protein supplementation is caused by the “time effect” or “total dose effect.” The results showed that pre-sleep protein supplementation has a better effect than evening protein intake. Second, we used the metabolic chamber to measure the TEE, SEE, and BEE. A high-precision metabolic chamber can simulate daily life in a free environment, and its use allowed precise measurement of EE, increasing the credible of our results. However, the study also has some limitations. We did not monitor energy intake in the free environment after the participants left the metabolic chamber the next day. Future studies should determine whether a subjective assessment of appetite after a pre-sleep protein supplementation would hinder or promote further energy intake the next morning or for a whole day in this population. In addition, we only focused on sedentary healthy people. The results of both present and previous studies indicate that metabolic responses to pre-sleep protein supplementation differ according to physical activity levels. Therefore, further research that involves a diverse population is needed to validate our findings. Finally, the long-term effects of pre-sleep protein supplementation were not evaluated and should be investigated in future studies.

## Conclusion

Pre-sleep protein supplementation combined with resistance exercise can significantly increase the SEE and fullness in the next morning after adjusting for the total 24-h energy intake in sedentary healthy adults. These effects have an obvious “time effect “of pre-sleep protein supplementation. These findings support that combining pre-sleep nutrition intervention with behavioral changes may improve sleep energy metabolism in the sedentary population.

## Data Availability Statement

The raw data supporting the conclusions of this article will be provided by the authors upon request.

## Ethics Statement

The studies involving human participants were reviewed and approved by the Ethics Committee of Shanghai University of Sport, Shanghai University of Sport. The patients/participants provided their written informed consent to participate in this study.

## Author Contributions

YH participated in the study design, subject recruitment, data collection, data processing, data analysis, and drafted the manuscript. XL participated in the design of the study and coordination and implementation of the experimental plan. ZZ contributed to the study design, acquisition of data, and critical revision. Z-BC conceived the study, participated in its design, helped to draft the manuscript, and contributed to the critical revision. All authors have read and approved the final version of the manuscript, and agreed with the order of presentation of the authors.

## Conflict of Interest

The authors declare that the research was conducted in the absence of any commercial or financial relationships that could be construed as a potential conflict of interest.

## Publisher’s Note

All claims expressed in this article are solely those of the authors and do not necessarily represent those of their affiliated organizations, or those of the publisher, the editors and the reviewers. Any product that may be evaluated in this article, or claim that may be made by its manufacturer, is not guaranteed or endorsed by the publisher.

## Footnotes

1
https://weixin.qq.com/
