# Supplementary material for: Pre-sleep Protein Supplementation Affects Energy Metabolism and Appetite in Sedentary Healthy Adults
Source: Front Nutr. 2022 Apr 28;9:873236. doi: 10.3389/fnut.2022.873236 (PMC9105224; doi:10.3389/fnut.2022.873236)
Supplement: Supplementary file 1 [file Table_1.docx]

Appendix1

Table1 Energy Expenditure, Substrate Oxidation Rate and Appetite Score during the experimental stage

|  | PLA | PRO-PLA | PLA-PRO | P-_model 1_ | P-_model 2_ |
| --- | --- | --- | --- | --- | --- |
| ***Energy Expenditure*** | | | | | |
| TEE (kcal/day) | 1883.1±293.4 | 1945.0±330.7**^a^** | 1938.6±317.4**^a^** | 0.002 | 0.677 |
| SEE (kcal/6h) | 296.6±46.6 | 318.3±44.3**^a^** | 329.9±45.2**^abc^** | 0.000 | 0.037 |
| BEE (kcal/day) | 1369.7±225.7 | 1408.3±268.4 | 1386.9±261.3 | 0.598 | 0.394 |
| ***Substrate oxidation rate(g/min)*** | | | | | |
| CHO oxidation rate_-24-h_ | 0.20±0.04 | 0.19[0.17, 0.23] | 0.20±0.04 | 0.388 | 0.378 |
| Fat oxidation rate_-24-h_ | 0.06[0.05,0.07] | 0.06[0.05, 0.08] | 0.06±0.01 | 0.991 | 0.974 |
| CHO oxidation rate_-Sleep_ | 0.06[0.05,0.08] | 0.08±0.02**^a^** | 0.08±0.02**^a^** | 0.003 | 0.388 |
| Fat oxidation rate_-Sleep_ | 0.06±0.01 | 0.06±0.02 | 0.07±0.01 | 0.627 | 0.682 |
| CHO oxidation rate_-BEE_ | 0.10±0.04 | 0.10±0.04 | 0.11±0.05 | 0.233 | 0.509 |
| Fat oxidation rate_-BEE_ | 0.06±0.02 | 0.06±0.02 | 0.06±0.02 | 0.494 | 0.516 |
| ***Appetite Score (mm)*** |  |  |  |  |  |
| Total appetite score | 56.9±19.2 | 53.4±19.5 | 51.9±20.5**^a^** | 0.044 | 0.654 |
| Desire to eat | 60[30,70] | 50[40,70] | 50.0±23.8 | 0.283 | 0.132 |
| Prospective consumption 52.6±22.8 | | 52.6±18.0 | 60[30,60] | 0.758 | 0.721 |
| Hunger | 53.5±22.0 | 49.0±22.4 | 50.3±22.7 | 0.497 | 0.617 |
| Fullness | 33.5±26.6 | 40[20,70] | 50[20,70] **^a, d^** | 0.042 | 0.038 |

Normally distributed data are represented by mean ± standard deviation (Mean ± SD), and non-normal distribution data are represented by the median and interquartile range.

a p<0.05 PLA-PRO, PRO-PLA VS PLA, b p<0.05 PLA-PRO VS PRO-PLA, adjusted for age, sex, %fat, relative VO2max, sleep quality, the time of sedentary behavior and moderate and vigorous intensity physical activity in the pre-experiment stage, resistance energy expenditure under each condition (model 1);

c p<0.05 PLA-PRO VS PRO-PLA, d p<0.05, PLA-PRO VS PLA additionally adjusted for 24-h total energy intake based on model 1 (model 2).
